# Effects of Mini-Dystrophin on Dystrophin-Deficient, Human Skeletal Muscle-Derived Cells

**DOI:** 10.3390/ijms21197168

**Published:** 2020-09-28

**Authors:** Jinhong Meng, John Counsell, Jennifer E. Morgan

**Affiliations:** 1 Dubowitz Neuromuscular Centre, Developmental Neuroscience Research and Teaching Department, UCL Great Ormond Street Institute of Child Health, 30 Guilford Street, London WC1N 1EH, UK; jinhong.meng@ucl.ac.uk (J.M.); j.counsell@ucl.ac.uk (J.C.); 2 NIHR Great Ormond Street Hospital Biomedical Research Centre, UCL Great Ormond Street Institute of Child Health & Great Ormond Street Hospital for Children NHS Foundation Trust, London WC1N 1EH, UK

**Keywords:** Duchenne muscular dystrophy, mini-dystrophin, lentivirus, promotor

## Abstract

Background: We are developing a novel therapy for Duchenne muscular dystrophy (DMD), involving the transplantation of autologous, skeletal muscle-derived stem cells that have been genetically corrected to express dystrophin. Dystrophin is normally expressed in activated satellite cells and in differentiated muscle fibres. However, in past preclinical validation studies, dystrophin transgenes have generally been driven by constitutive promoters that would be active at every stage of the myogenic differentiation process, including in proliferating muscle stem cells. It is not known whether artificial dystrophin expression would affect the properties of these cells. Aims: Our aims are to determine if mini-dystrophin expression affects the proliferation or myogenic differentiation of DMD skeletal muscle-derived cells. Methods: Skeletal muscle-derived cells from a DMD patient were transduced with lentivirus coding for mini-dystrophins (R3–R13 spectrin-like repeats (ΔR3R13) or hinge2 to spectrin-like repeats R23 (ΔH2R23)) with EGFP (enhanced green fluorescence protein) fused to the C-terminus, driven by a constitutive promoter, spleen focus-forming virus (SFFV). Transduced cells were purified on the basis of GFP expression. Their proliferation and myogenic differentiation were quantified by ethynyl deoxyuridine (EdU) incorporation and fusion index. Furthermore, dystrophin small interfering ribonucleic acids (siRNAs) were transfected to the cells to reverse the effects of the mini-dystrophin. Finally, a phospho-mitogen-activated protein kinase (MAPK) array assay was performed to investigate signalling pathway changes caused by dystrophin expression. Results: Cell proliferation was not affected in cells transduced with ΔR3R13, but was significantly increased in cells transduced with ΔH2R23. The fusion index of myotubes derived from both ΔR3R13- and ΔH2R23 -expressing cells was significantly compromised in comparison to myotubes derived from non-transduced cells. Dystrophin siRNA transfection restored the differentiation of ΔH2R23-expressing cells. The Erk1/2- signalling pathway is altered in cells transduced with mini-dystrophin constructs. Conclusions: Ectopic expression of dystrophin in cultured human skeletal muscle-derived cells may affect their proliferation and differentiation capacity. Caution should be taken when considering genetic correction of autologous stem cells to express dystrophin driven by a constitutive promoter.

## 1. Introduction

Transplantation of autologous, skeletal muscle-derived stem cells that have been genetically corrected to express dystrophin is a promising strategy to treat Duchenne muscular dystrophy (DMD). Dystrophin is a large protein that is expressed primarily in the muscles and brain [1]. In skeletal muscles, dystrophin is mainly located at the inner layer of the sarcolemma of muscle fibres, connecting the intracellular compartment with the extracellular matrix proteins to stabilize the sarcolemma and protect the myofiber from disruption during contraction [2,3]. Dystrophin is also expressed in activated satellite cells, where it regulates satellite cell polarity and asymmetric division [4]. Dystrophin contains multiple functional motifs, which play complex but important roles via their interaction with members of the dystrophin-associated protein complex (DAPC) [5,6], neuronal nitric oxide synthase (nNOS) [7,8], intracellular cytoskeleton molecules [9,10], or Par1b, a regulator of cell polarity [4]. Interestingly, dystrophin is switched off when satellite cells become myoblasts, and on again when they differente into myofibres [4], suggesting that dystrophin protein is not required when cells are at the myoblast stage. However, in some gene therapy scenarios, in order to deliver a dystrophin construct containing the majority of functional motifs to either skeletal muscle stem cells [11,12] ex vivo, or into muscles of animal models of DMD via direct injection [13], small but ubiquitously-expressed promoters have usually been used. Dystrophin would consequently be expressed in cells at all stages of myogenic differentiation, including proliferating muscle cells. This has raised concerns that upregulation of mini-dystrophin may change the performance of the cells, given that the isoforms of mini-dystrophin usually contain functional motifs that can elicit signalling pathways via their binding to corresponding intracellular or cell membrane components. This may eventually lead to unexpected outcomes in downstream application of the cells. 

The aim of this study is to determine the effects of mini-dystrophin expression on the function of DMD muscle derived-cells, a promising autologous cell type for stem cell therapy to treat DMD [12,14]. These cells are highly myogenic in vitro and have contributed to muscle regeneration once transplanted into immunodeficient mouse models [14,15]. They can be transduced with lentivirus coding for mini-dystrophins R3–R13 spectrin-like repeats (ΔR3R13) or hinge2 to spectrin-like repeats R23 (ΔH2R23), and following their intra-muscular transplantation into mdx nude mice [12], contribute to regenerated myofibres within which mini-dystrophin is expressed. However, in this study, the mini-dystrophins were driven by either a spleen focus-forming virus (SFFV) [12] or a human desmin promoter [12,16,17], both of which were strong and active in proliferating cells and differentiated myotubes. It is not clear whether the over-expression of mini-dystrophin in non-differentiated cells would affect their ability to either proliferate or undergo myogenic differentiation. To address this issue, we purified the mini-dystrophin-transduced, DMD muscle-derived cells on the basis of GFP expression and examined their proliferation and myogenic differentiation in culture in comparison to non-transduced cells. The outcomes of the study provide evidence for the first time that upregulation of dystrophin in proliferating muscle cells does change their behaviour in an isoform-dependent manner.

## 2. Results

### 2.1. Lentiviral Vectors Coding for Different Mini-Dystrophins Driven by Different Promoters

**Figure 1:**
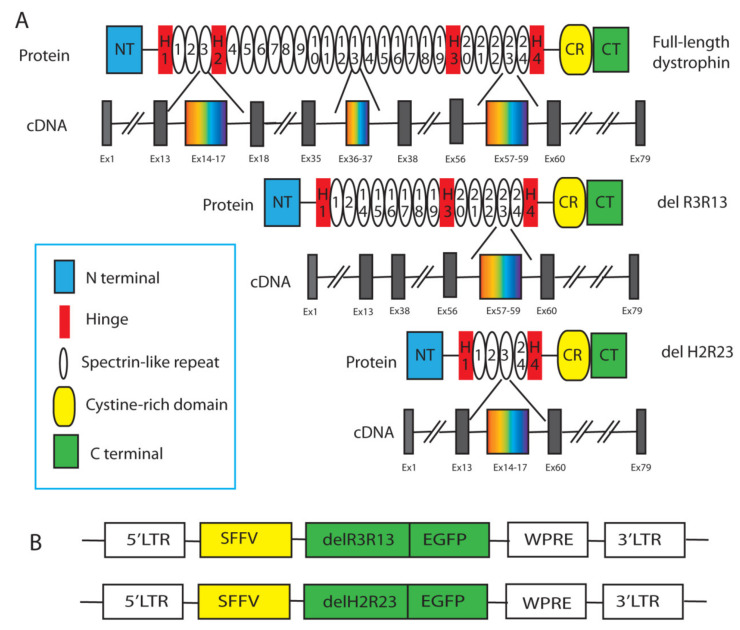
Schematic illustration of lentiviral transfer plasmid (**A**) and the protein and complementary (c)DNA structure (**B**) of the mini-dystrophins used in this study.

Two mini-dystrophin constructs, lacking the R3–R13 spectrin-like repeats (ΔR3R13) or hinge2 to spectrin-like repeats R23 (ΔH2R23), respectively, were used in this study [12]. Correspondingly, the constructs lack either dystrophin Exon 14-37 (ΔR3R13) or Exon 18-59 (ΔH2R23) in their cDNA sequence (Figure 1). The size of the cDNA of ΔR3R13 is 7.4 Kb, and ΔH2R23 is 4.2 Kb. Both constructs have an enhanced green fluorescent protein (EGFP) (720bp) cassette fused to the C-terminus of the dystrophin cassette, to facilitate the purification of the transduced cells. These constructs were driven by a ubiquitous SFFV (412bp) promoter (Figure 1). The size of the mini-dystrophin–EGFP inserts were 8.1 Kb (ΔR3R13-EGFP) and 5.0 Kb (ΔH2R23-EGFP), respectively. The size of the integrated provirus in the cell genome was 11.2 Kb (ΔR3R13–EGFP) and 8.1 Kb (ΔH2R23–EGFP) (Figure 1).

### 2.2. Transduction and Enrichment of Mini-Dystrophin–GFP+ Cells

**Figure 2:**
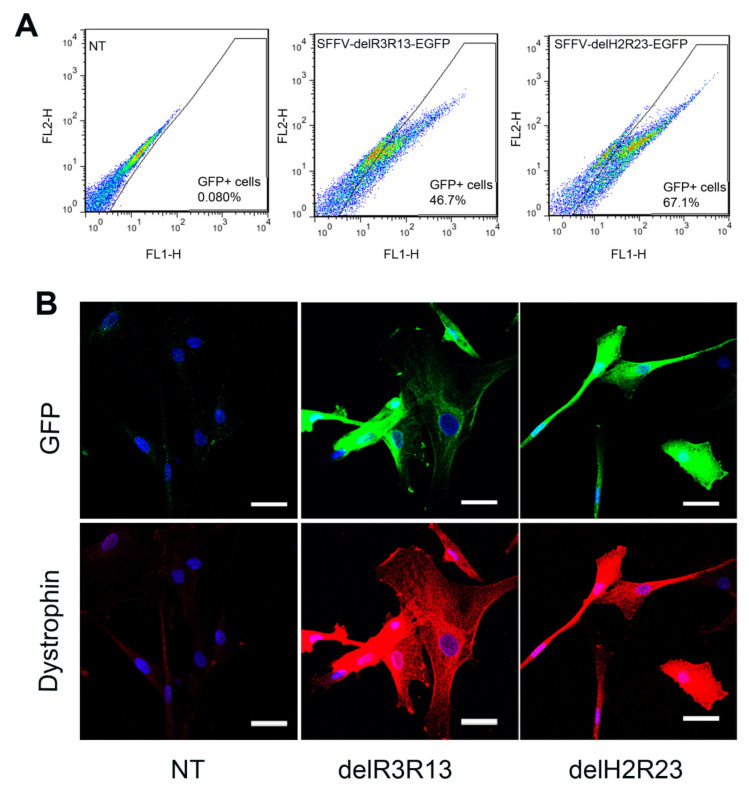
Fluorescence-activated cell sorting (FACS) analysis of the transduced cells (**A**) and immunostaining of GFP (green) and dystrophin (red) of non-transduced, R3–R13 spectrin-like repeats (ΔR3R13)–GFP or hinge2 to spectrin-like repeats R23 (ΔH2R23)–GFP transduced cells (**B**). Nuclei were stained with 4′,6-diamidino-2-phenylindole (DAPI) (blue). Scale bar = 30 µm.

Skeletal muscle-derived cells, isolated from a DMD patient with a deletion of exons 45–50 in the dystrophin gene (ΔEx45-50) [14], were transduced with lentiviruses (LVs) at multiplicity of infection (MOI) 5. Transduction efficiencies were determined 5 days after transduction. As the mini-dystrophins were fused to an EGFP cassette, GFP was used as surrogate marker for dystrophin expression in transduced cells. Fluorescence-activated cell sorting (FACS) analysis showed that the percentage of GFP+ cells in each transduced group was 46.7% (SFFV–ΔR3R13–EGFP) and 67.1% (SFFV–ΔH2R23–EGFP) (Figure 2A), which is highly dependent on the size of the provirus, as previously described [12]. To investigate the effects of the dystrophin on cells, GFP+ cells were purified and expanded for downstream analysis.

Immunostaining of dystrophin on purified cells, which were cultured in proliferation medium, showed both mini-dystrophin ΔR3R13–EGFP- and ΔH2R23–EGFP-transduced cells expressing dystrophin and GFP, while non- transduced cells were negative for both GFP and dystrophin (Figure 2B).

### 2.3. Proliferation of DMD Muscle–Derived Cells after Lentiviral Transduction

**Figure 3:**
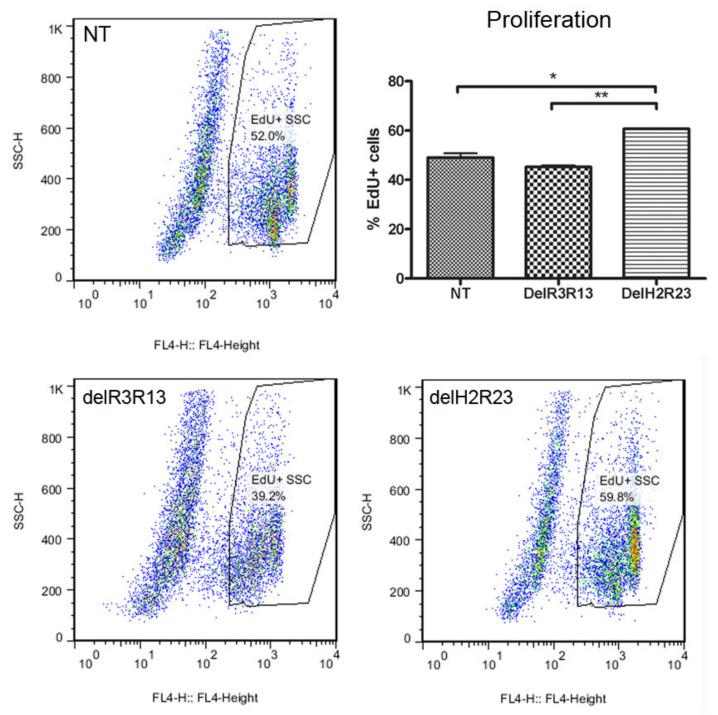
Effects on cell proliferation after cells were transduced with mini-dystrophin lentivirus driven by the spleen focus-forming virus (SFFV) promoter. Cells transfected with mini-dystrophin ΔR3R13 had no effect on cell proliferation, while ΔH2R23 significantly increased the proliferation of human muscle-derived cells. * *p* < 0.05; ** *p* < 0.01.

To determine whether the expression of mini-dystrophin in muscle-derived cells would affect their proliferation, we performed EdU analysis on GFP+ LV-transduced cells expanded in vitro, at equivalent mean population doublings (mpds) (14.56–19.87), using non-transduced cells as a control. The percentage (mean ± SEM) of EdU+ cells was 49.02% ± 1.78% for non-transduced cells, 45.22% ± 0.68% for SFFV–ΔR3R13 transduced cells and 60.68% ± 0.24% for SFFV–ΔH2R23 transduced cells (*n* = 6 for each group). There was a significantly higher percentage of EdU+ cells in ΔH2R23–EGFP (*p* < 0.001) transduced cells, but not ΔR3R13–EGFP transduced cells (*p* > 0.05), in comparison to non-transduced cells (Figure 3).

To further confirm the effect of the mini-dystrophins on the proliferation of human muscle-derived cells, we constructed lentiviral vectors expressing either ΔR3R13–EGFP or ΔH2R23–EGFP driven by another constitutive promoter, the human desmin (hDes-) promoter, and transduced the cells in the same way as we did with the SFFV-driven lentiviral vectors. When we performed the proliferation assay with the purified GFP+ cells from cells that had been transduced with hDes–LVs, we found that hDes–ΔH2R23–EGFP cells (79.47% ± 1.48%; *n* = 3; *p* < 0.001) contained significantly more EdU+ cells than non-transduced cells (59.47% ± 0.33%; *n* = 3); while there was no difference in the % of EDU+ cells transduced with hDes–ΔR3R13–EGFP (62.13% ± 0.78%, *n* = 3) compared to non-transduced cells (*p* > 0.05) (Supplementary Figure S1).

These data show that the proliferation of DMD muscle-derived cells is significantly increased by the overexpression of ΔH2R23–EGFP, but not ΔR3R13–EGFP, regardless of the promoter used. In subsequent experiments, we only used cells in which the inserted gene was driven by the SFFV promoter.

### 2.4. Effects of Mini-Dystrophin on the Myogenic Differentiation of DMD Muscle Pericytes

**Figure 4:**
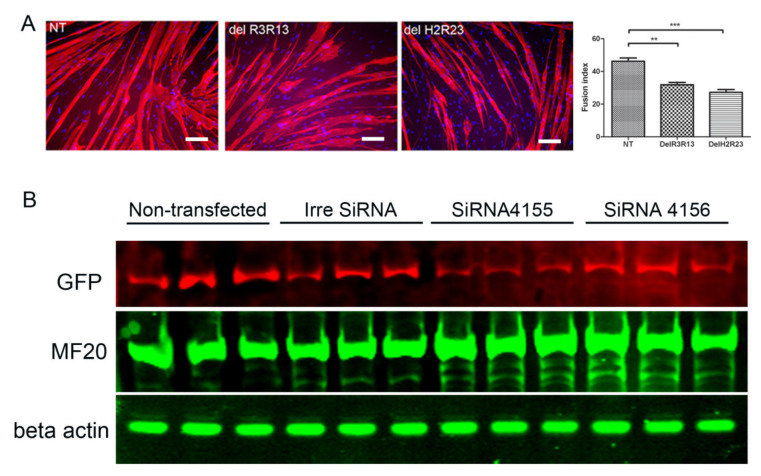

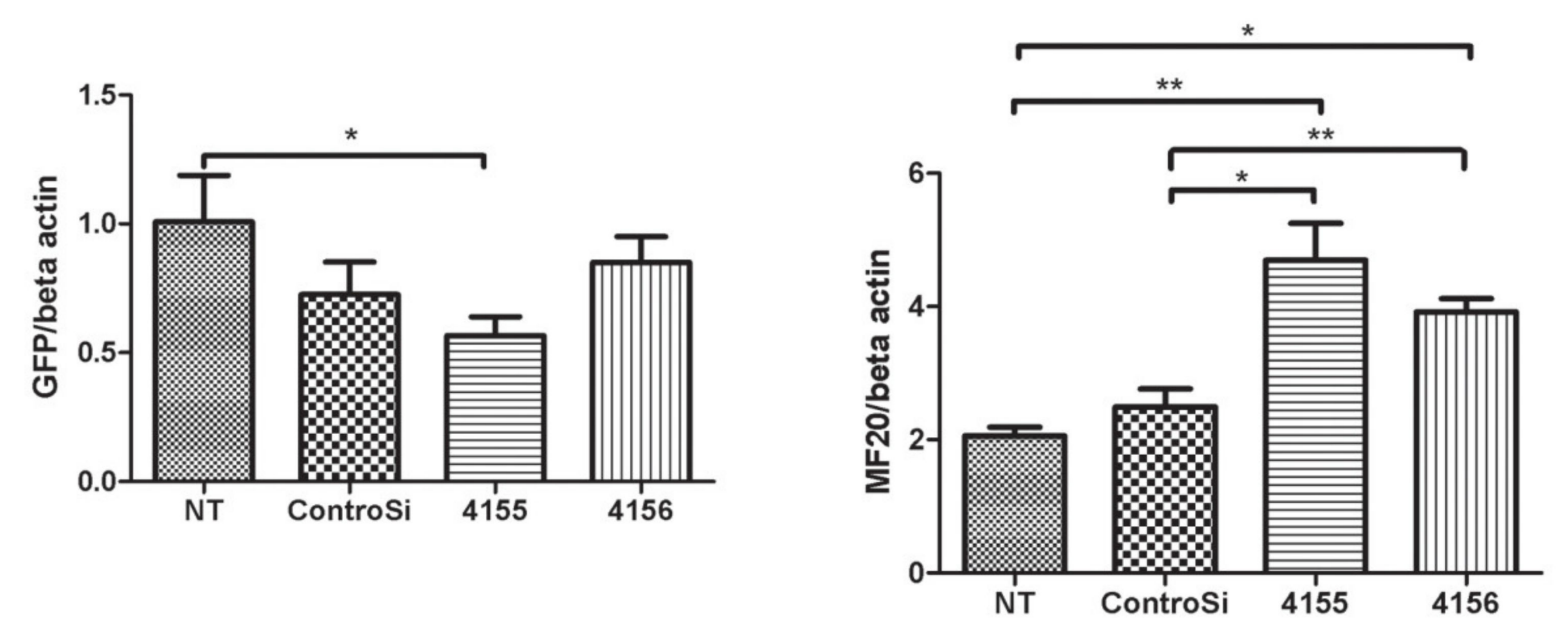
Mini-dystrophins reduce the terminal myogenic differentiation of human Duchenne muscular dystrophy (DMD) muscle cells (**A**), and this is reversed by the knockdown of dystrophin expression using siRNAs (**B**). Mini-dystrophin GFP+ cells were induced to undergo myogenic differentiation for 7 days, and the fusion index (FI) of each experimental group showed that both ΔR3R13–GFP and ΔH2R23–GFP transduced cells have a reduced fusion index compared to non-transduced cells (**A**), scale bar = 25 µm. To determine whether the effect of mini-dystrophin on myogenic differentiation is reversible, ΔH2R23–GFP cells were transfected with one of the two different dystrophin siRNAs (4155 and 4156), and then induced to undergo myogenic differentiation for 7 days. Western blot showed that after SiRNA transfection, the dystrophin ΔH2R23–GFP expression was reduced and myosin heavy chain increased in cells transfected with siRNA 4155, but not in cells transfected with siRNA 4156, (**B**). NT: non-transfected SFFV–ΔH2R23–GFP cells; ConSi, 4155, 4156: SFFV–ΔH2R23–GFP cells transfected with control siRNA or dystrophin siRNAs 4155 or 4156, respectively. * *p* < 0.05; ** *p* < 0.01.

To determine the effects of mini-dystrophin on the terminal myogenic differentiation of DMD muscle-derived cells, GFP+ cells from SFFV–LV transduced cells were induced to undergo myogenic differentiation and stained with an antibody to myosin heavy chain, and their fusion indices compared. The fusion index of non-transduced, LV–SFFV–ΔR3R13–EGFP and LV–SFFV–ΔH2R23–EGFP transduced cells were 46.2% ± 1.95%, 31.9% ± 1.4%, and 27.2% ± 1.8% (mean ± SEM), respectively (Figure 4). The fusion indices of LV–SFFV–ΔR3R13–EGFP (*p* < 0.01) or LV–SFFV–ΔH2R23–EGFP (*p* < 0.001) transduced cells were significantly lower than non-transduced cells, suggesting that the mini-dystrophin isoforms ΔR3R13 and ΔH2R23 compromised the myogenic differentiation of the DMD muscle cells.

To determine whether this adverse effect was indeed caused by the overexpression of dystrophin and whether it could be reversed, we repressed dystrophin expression by siRNA treatment, using cells that have been transduced with ΔH2R23, whose myogenic capacity was affected more severely than cells transduced with ΔR3R13.

GFP+ cells that had been transduced with ΔH2R23 were transfected with siRNA binding to either dystrophin exon 63–64 (siRNA 4155) or exon 67 (siRNA 4156). The cells were then induced to undergo myogenic differentiation for 7 days before assessing dystrophin knockdown, quantified by the intensity of its surrogate marker, GFP [12], and the extent of myogenesis, indicated by the level of myosin heavy chain by western blot. After siRNA transfection, the dystrophin ΔH2R23 expression was significantly reduced in cells transfected with siRNA 4155 (*p* < 0.05), but not in cells transfected with siRNA 4156, in comparison to those transfected with the control siRNA (Figure 4). Interestingly, MF20, which is expressed during myogenic differentiation, was significantly increased in cells transfected with both siRNA 4155 (*p* < 0.01) and 4156 (*p* < 0.05), in comparison to non-transfected cells. These data suggest that the defects in myogenic differentiation caused by mini-dystrophin ΔH2R23 being produced prior to terminal differentiation can be reversed by knocking down its expression by siRNA silencing.

### 2.5. Signalling Pathway Affected by Dystrophin Expression

**Figure 5:**
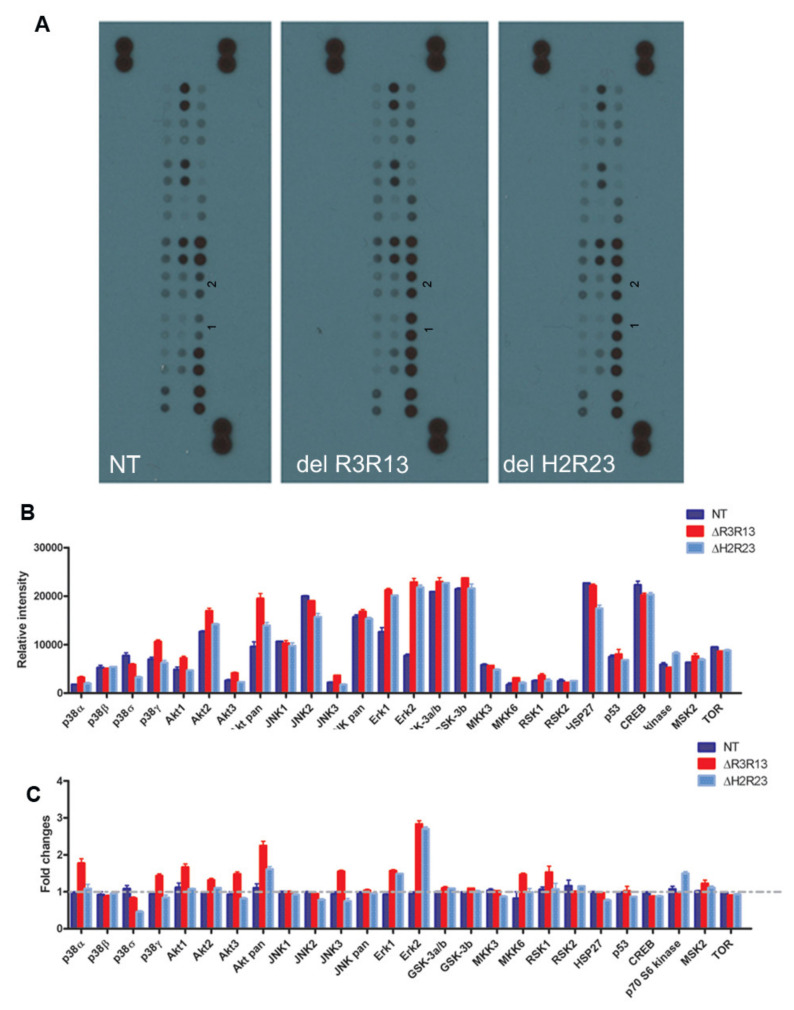
Signalling pathways involved in the effect of the mini-dystrophins on DMD muscle- derived cells. Non-transduced cells (NT), GFP+ cells purified from LV–SFFV–ΔR3R13–GFP or LV–SFFV–ΔH2R23–GFP-transduced cells were expanded and analysed with a human phosphor-MAPK array kit (**A**). Data shown are from a 1 min exposure to X-ray film. 1 and 2 in each blot denote the *phosph-Erk2* and *phosph-Erk1* signals respectively. Relative intensity measurements show expression levels of the 26 MAPKs in each sample (**B**), as well as by fold changes of each kinase against non-transduced cells in experimental groups (**C**).

We have shown that overexpression of mini-dystrophin adversely affects cell proliferation and myogenic differentiation. Next, we wished to explore the underlying mechanisms involved, which may be targeted to improve the therapeutic potential of the dystrophin-transduced cells in treating DMD. Multiple intracellular signalling pathways play roles in muscle cell proliferation and myogenic differentiation [18,19,20,21,22,23,24,25,26,27,28,29,30,31]. These include the extracellular signal-regulated kinase (ERK)–MAPK, p38 MAPK, and phosphatidylinositol 3-kinase (PI3K)/AKT pathways. We performed a MAPK array assay to determine the cellular signalling pathways involved, by screening the changes in phosphorylated MAPK in non-transduced, ΔR3R13–EGFP or ΔH2R23–EGFP transduced cells (Figure 5). The level of phospho-MAPKs varies within each cell group (Figure 5A,B), with a higher expression of kinases involved in the PI3/Akt-, JNK-, ERK-, GSK-, HSP27-, and CREB- pathways than other kinases, determined by the relative intensity of the protein in the same samples. However, when we compared the relative intensity (fold change) of each phospho- protein among cell groups, we found that, in comparison to non-transduced cells, the major differences between ΔR3R13–EGFP/ΔH2R23–EGFP transduced cells and non-transduced cells were the higher levels (1.5–3.0 fold change) of phosphor-Erk1/2 and pan-Akt. In addition, the ΔR3R13–EGFP cells contained higher levels of p38α, p38γ, Akt1, Akt3, JNK3, MKK6, and RSK1, while the ΔH2R23–EGFP expressing cells contained at least 1.5-fold more P70 S6 kinase than other groups. Both ΔR3R13–EGFP- and ΔH2R23–EGFP-expressing cells contained decreased p38δ protein, with ΔH2R23–EGFP cells having the lowest expression.

## 3. Discussion

In our study, we lentivirally transduced DMD skeletal muscle-derived cells with two different isoforms of mini-dystrophin, driven by constitutive promoters that are active in proliferating myoblasts [14]. Transduced cells were purified by FACS based on their expression of GFP, a surrogate marker fused to the C-terminus of the dystrophin construct. We found that both the cell proliferation and differentiation capacity changed in transduced cells in an isoform-dependent manner. Proliferation of the muscle cells was significantly affected by mini-dystrophin ΔH2R23 (*p* < 0.001) but not ΔR3R13 (*p* > 0.05) expression (Figure 3). Both isoforms caused decreased myogenic differentiation of the cells, with the least differentiation in ΔH2R23-expressing cells (Figure 4). Interestingly, the effect of dystrophin overexpression on myogenesis was reversible when dystrophin was silenced using SiRNA treatment (Figure 4).

Dystrophin exerts its function through interactions with other cellular ligands, mediated by the functional motifs of the protein. Different isoforms of dystrophin affect the properties of the cells differently, due to the nature of the motifs they contain. Both of the mini-dystrophin isoforms (ΔR3R13 and ΔH2R23) contain the intact N-terminal actin binding domain 1 (ABD1), cysteine-rich (CR) domain, and carboxy-terminal (CT) domains (Figure 1). This means that in the transduced muscle cells, mini-dystrophin has the ability to bind intracellular F-actin, β-dystroglycan, and syntrophins/α-dystrobrevin via its ABD1, CR, and CT domains, respectively. The ABD1 structure has been shown to be related to the stability of dystrophin proteins and binding affinity to intracellular actin [32,33]. β-dystroglycan is an important cell adhesion receptor, whose cell membrane location relies on the presence of dystrophin [34,35]. The binding of the mini-dystrophins to both F-Actin and β-dystroglycan links the intracellular skeleton and the extracellular matrix, strengthening the stability of the cell membrane. In addition to the crucial structural role at the plasma membrane, β-dystroglycan can be a scaffold for various signalling cascades, including the extracellular signal-related, kinase-mitogen-activated protein kinase (ERK–MAPK) [21,28]. This is in line with our finding that cells expressing either of the dystrophin isoforms have increased Erk1/2 activity, in comparison to non-transduced cells (Figure 5). ERK activation is indispensable for cell proliferation [31] and differentiation [18,27]. Sustained activation of Erk1/2 is necessary for G1- to S-phase progression and is associated with the inactivation of anti-proliferative genes. Stimulation of C2C12 mouse myoblasts with leukemia inhibitory factor (LIF) led to increased proliferation and dramatically decreased myogenic differentiation via the ERK signalling pathway. Erk1/2 activation is also involved in myostatin-induced defects in myogenesis [18]. Our data suggest that overexpression of mini-dystrophin in DMD muscle-derived cells affects either their proliferation or their differentiation by recruiting beta-dystroglycan to the cell membrane, which further triggers the downstream Erk1/2 signalling pathway.

Restoration of the dystrophin-associated complex (DAPC) is a readout of the functionality of the restored dystrophin in myofibres [36,37,38]. We have previously investigated the restoration of the DAPC on donor-derived myofibres in mdx nude mice muscle that had been transplanted with mini-dystrophin (ΔR3R13 and ΔH2R23)-expressing muscle cells. Both isoforms, ΔR3R13 and ΔH2R23, recruited members of the DAPC (including α-sarcoglycan, β-dystroglycan, and γ-sarcoglycan) to the sarcolemma of the mini-dystrophin-expressing myofibres; however, only ΔR3R13, which contains the nNOS binding site, could recruit nNOS to the membrane of the myofibres [12].

We showed that myogenic differentiation increased in cells treated with either of the different siRNAs to target either dystrophin exon 63–64 (SiRNA 4155) or exon 67 (SiRNA 4156), although mini-dystrophin was significantly reduced only in cells treated with siRNA 4155. It is not clear why the silencing efficiency differed between the two siRNAs, with siRNA 4155 being more effective. It is known that some siRNA sequences may be less effective than others, due to the three-dimensional (3D) conformation of the mRNAs and the accessibility of the siRNA to its target sequence [39,40,41,42]. Although both siRNAs would be expected to silence endogenous Dp71, as well as the mini-dystrophin, we did not quantify the Dp71 protein. Dp71 has been shown to have distinct subcellular distribution from that of Dp427m [43,44], and exerts unique and multifunctional roles, including cell adhesion, water homeostasis, cell division, and nuclear architecture. However, there is no evidence that Dp71 affects the myogenic differentiation of skeletal muscle cells. Besides, whether the level of Dp71 actually increases [45] or decreases [46] during myogenic differentiation is matter of debate. It is possible that knocking down Dp71 may have contributed to the increased myogenicity of the cells in our experiments, but any possible role of Dp71 in myogenic differentiation would be the subject of future work.

Our data also showed that the effects of the mini-dystrophin on DMD muscle-derived cells are isoform-dependent. Cell proliferation was significantly affected in cells expressing ΔH2R23, but not ΔR3R13 dystrophin, while differentiation was significantly affected by either ΔH2R23 or ΔR3R13 dystrophin expression. This could be explained by the fact that the isoforms contained different functional motifs. The major difference between the two isoforms is the presence of spectrin-like repeats R16–17, which are an nNOS binding site [8], in ΔR3R13 but not ΔH2R23. nNOS is an enzyme that catalyses the production of nitric oxide (NO) from L-arginine [47]. NO is an important modulator [48] of skeletal muscle vasomodulation [49,50], contractility [51], mitochondrial respiration, carbohydrate metabolism [52], and neuromuscular transmission. Aberrant nNOS signalling can negatively impact important clinical features of dystrophinopathies and sarcoglycanopathies [53,54]. Our data show that with the presence of the nNOS binding domain, the mini-dystrophin ΔR3R13–GFP expressing cells maintain a normal proliferation rate, accompanied by higher levels of p38α, p38γ, Akt1, Akt3, JNK3, MKK6, and RSK1.

Interestingly, while both ΔR3R13–GFP and ΔH2R23–GFP cells contained elevated Erk1/2, only ΔH2R23–GFP cells had significantly increased proliferation. It is noticeable that in ΔH2R23–GFP expressing cells, p70S6K is also upregulated. P70S6K has been shown to be necessary for progression through the cell cycle, specifically for the G_1_ to S phase transition [23,55]. It is possible that only the additive effects of these two signalling pathways are responsible for increased cell proliferation.

In summary, our study provides evidence that overexpression of mini-dystrophin in muscle cells compromises their myogenic capacity. This deleterious effect may or may not be accompanied by a proliferation defect, depending on the isoform of the dystrophin expressed. Multiple signalling pathways, such as β-dystroglycan and nNOS, as well as Erk1/2–, p70S6K–, AKT–, or p38–MAPK pathways, may be involved in underlying mechanisms. Further studies to validate these pathways are needed to investigate the exact mechanism of action of mini-dystrophin isoforms on DMD cells. Our observations raise concerns for gene therapy strategies using a ubiquitous promoter to restore dystrophin to either cells or tissues, which may introduce deleterious effects. A muscle-specific promoter would provide an ideal alternative to target myotubes/myofibres where dystrophin is predominantly expressed.

Indeed, with the development of gene therapy clinical trials, the use of constitutive promoters, such as cytomegalovirus (CMV) promoter (clinical trial no. NCT02354781 [56]), has been replaced by muscle specific promoters, such as MHCK7 (clinical trial no. NCT03375164) [57], and muscle hybrid promoters [58], in order to restrict the expression of mini- or micro- dystrophin to skeletal muscle and heart tissues.

## 4. Materials and Methods

### 4.1. Ethics

This work was performed under the National Health Service (NHS) National Research Ethics: setting up of a rare diseases biological samples bank (biobank) for research to facilitate pharmacological, gene, and cell therapy trials in neuromuscular disorders (Research Ethics Committee (REC) reference number: 06/Q0406/33, date of approval: 17 July 2006), as well as the use of cells as a model system to study pathogenesis and therapeutic strategies for neuromuscular disorders (REC reference 13/LO/1826, date of approval: 27 September 2013).

### 4.2. Production of Lentiviral Vectors and Transduction of DMD Human Muscle-Derived Cells

LV–SFFV–ΔR3R13–EGFP, LV–SFFV–ΔH2R23–EGFP, LV–hDesmin–ΔR3R13–EGFP, and LV–hDesmin–ΔH2R23–EGFP was produced by co-transfecting the transfer plasmid, packaging plasmids (pMDLg/pRRE and pRSV–Rev) and the envelope plasmid (pMD2.G) at a ratio of 4:2:1:1 in HEK293T cells. Supernatant was collected 48 and 72 h after transfection, and concentrated by ultracentrifugation at 23,000× *g* for 2 h at 4 °C. Concentrated LVs were stored at −80 °C.

LV titre was determined with HEK293T cells. HEK293T cells were plated at 1 × 10^4^/well in 24-well plates overnight before being transduced with different amounts (1 µL, 5 µL, 10 µL, and 20 µL) of lentiviruses. Cells were changed into fresh medium 6 h after adding the virus. The transduced cells were then expanded in 10% fetal bovine serum (FBS) containing DMEM medium for 2–3 passages before the extraction of genomic DNA and qPCR to determine the viral copy number/cell (VCN/cell) within the transduced cells. The primers and probes used for qPCR were the woodchuck hepatitis virus posttranscriptional regulatory element (WPRE)-forward primer: TGGATTCTGCGCGGGA; WPRE-reverse: GAAGGAAGGTCCGCTGGATT; WPRE-probe: CTTCTGCTACGTCCCTTCGGCCCT; β-actin-forward: CAGCGGAACCGCTCATTGCCAATGG; β-actin-reverse: TCACCCACACTGTGCCCATCTACGA; and β-actin-probe: ATGCCCTCCCCCATGCCATCCTGCGT. The titre (transduction unit (TU)/mL) of the LV was calculated as the VCN/cell × the number of cells seeded/volume (µL) of concentrated LV × 1000.

DMD muscle-derived cells [14] were maintained in M10 medium in collagen I (Sigma, Dorset, UK)-coated 75 cm^2^ flasks at a density of 2.5 × 10^5^ cells/flask. Cells at mean population doublings (mpds) of 14.56–19.87 were used in this study. For LV transduction, 1 × 10^5^ cells were transduced with LV at MOI 5 for 6 h before changing to fresh medium. Transduced cells were expanded for 5 days, and GFP+ cells were purified by FACS sorting using a Moflo XPD cell sorter (Beckman Coulter, High Wycombe, UK) for downstream analysis.

### 4.3. EdU Assay

Cells were plated at 1 × 10^5^/wells on collagen I-coated six-well plates and incubated in 10 µM EdU containing M10 medium for 16 h before being processed for proliferation assay using a Click-iT Plus EdU Alexa Fluor 647 Flow Cytometry Assay Kit (Thermo Fisher, Paisley, UK), following the manufacturers’ instructions. Non-transduced cells were used as a control. The percentage of EdU+ cells was determined using a BD FACSCalibur machine (BD Biosciences, San Jose, CA, USA).

### 4.4. Myogenic Differentiation

Cells were plated either at 5 × 10^4^ cells/well on 0.1 mg/mL Matrigel (BD Biosciences, San Jose, CA)-coated, eight-well Nunc Lab-Tek Chamber Slides (Sigma, Dorset, UK), or at 5 × 10^5^ cells/well on Matrigel coated six-well plates in M10 medium. Twenty-four hours later, cells were changed into M2 differentiation medium (Megacell DMEM containing 2% FBS) for myogenic differentiation for 7 days.

### 4.5. Immunostaining of Myotubes

Differentiated cells on eight-well chamber slides were fixed with 4% paraformaldehyde for 15 min at room temperature, and immunostained with an antibody against myosin heavy chain (MF20; Developmental Studies Hybridoma Biobank (DSHB), Iowa, USA, 1:500) for 2 h, followed by goat anti-mouse IgG (H+L) (Thermo Fisher, Paisley, UK; 1:1000) for 1 h. Nuclei were counterstained with 10 µg/mL 4′,6-diamidino-2-phenylindole (DAPI). Fusion indices were calculated as the percentage of the nuclei that were within myotubes (MF20+ and containing three or more nuclei). For quantification, five fields per well were randomly chosen, and three wells per group were counted.

### 4.6. SiRNA Transfection

GFP+ cells were plated at 2 × 10^5^ cells/well or 2.5 × 10^4^ cells/well in Matrigel-coated six-well plates or eight-well chamber slides, respectively. On the day of transfection, LipofectAMINE 2000 (Thermo Fisher, Paisley, UK) and 100 nM dystrophin or irrelevant siRNAs (Thermo Fisher, Paisley, UK) were pre-diluted in OptiMEM (Thermo Fisher, Paisley, UK) and mixed to form complexes at room temperature (RT) for 25 min before being added to cells. Dystrophin siRNAs 4155 and 4156 was designed to target the exon 63–64 and exon 67 of the dystrophin gene, respectively. SiRNA transfection was performed twice (24 h and 72 h after plating) to enhance their knockdown efficiency, and the medium was changed into differentiation medium after the second transfection. Protein lysates were collected 7 days after differentiation for Western blot analysis.

### 4.7. Western Blot

Differentiated cells were lysed with radio-immunoprecipitation assay (RIPA) buffer (Sigma, Dorset, UK), supplemented with protease inhibitor (Roche, Welwyn Garden City, UK) on ice for 15 min. The cell lysate was collected and boiled for 5 min before being centrifuged at 14,000× *g* for 10 min at 4 °C. Protein concentration was determined using a Pierce BCA Protein Assay Kit (Thermo Fisher, Paisley, UK). A total of 30 µg/well of each sample was loaded onto NuPAGE Novex 10% Bis-Tris gel (Thermo Fisher, Paisley, UK), and proteins were separated at a constant voltage of 150 V for 1.5 h, before being transferred to a nitrocellulose membrane using at constant current of 300 mA for 2 h. The membrane was blocked with Odyssey block solution (LI-COR Biosciences, Cambridge, UK) for one hour, and then incubated with primary antibodies against GFP (rabbit polyclonal IgG (H+L), 1:2000; Thermo Fisher, Paisley, UK), MF20 (mouse monoclonal IgG2b, 1:1000; DSHB, Iowa, USA), using beta actin (mouse monoclonal IgG1, 1:5000; Sigma, Dorset, UK) as a control. After washing with PBS containing 0.1% Tween 20 (PBST) for 15 min × 3 times at room temperature, the membrane was incubated with IRDye 680RD goat anti-rabbit and IRDye 800CW goat anti-mouse second antibodies (1:15,000, LI-COR Biosciences, Cambridge, UK) for 1 h at RT. The image of the blotted membrane was acquired by an Odyssey Clx infrared imaging system (LI-COR Biosciences, Cambridge, UK) using Image Studio Lite 5.2 software.

### 4.8. MAPK Signalling Pathway Assay

Each group of cells was expanded in M10 medium, and protein lysates were collected from proliferating cells at equivalent mpds. The level of the signalling molecules within the MAPK pathway in these samples was compared using the Human Phospho-Mitogen-Activated Protein Kinase (MAPK) Antibody Array kit (R&D Systems, Abingdon, UK), in which an array of phospho-specific antibodies was spotted in duplicate on nitrocellulose membranes. Cell lysates were diluted and mixed with a cocktail of biotinylated detection antibodies; the sample/antibody mixture was then incubated with the array. Streptavidin–horseradish peroxidase and chemiluminescent detection reagents were then added, and a chemiluminescent signal was produced in proportion to the amount of phosphorylated protein bound. The intensity of the signal of each protein was measured using ImageJ software.

## Supplementary Materials

The following are available online at https://www.mdpi.com/1422-0067/21/19/7168/s1, Figure S1: Effects on cell proliferation after cells were transduced with mini-dystrophin lentivirus driven by the human desmin (hDes) promoter.
