# Supplementary material for: Effects of Mini-Dystrophin on Dystrophin-Deficient, Human Skeletal Muscle-Derived Cells
Source: Int J Mol Sci. 2020 Sep 28;21(19):7168. doi: 10.3390/ijms21197168 (PMC7582244; doi:10.3390/ijms21197168)
Supplement: Supplementary file 1 [file ijms-21-07168-s001.pdf]

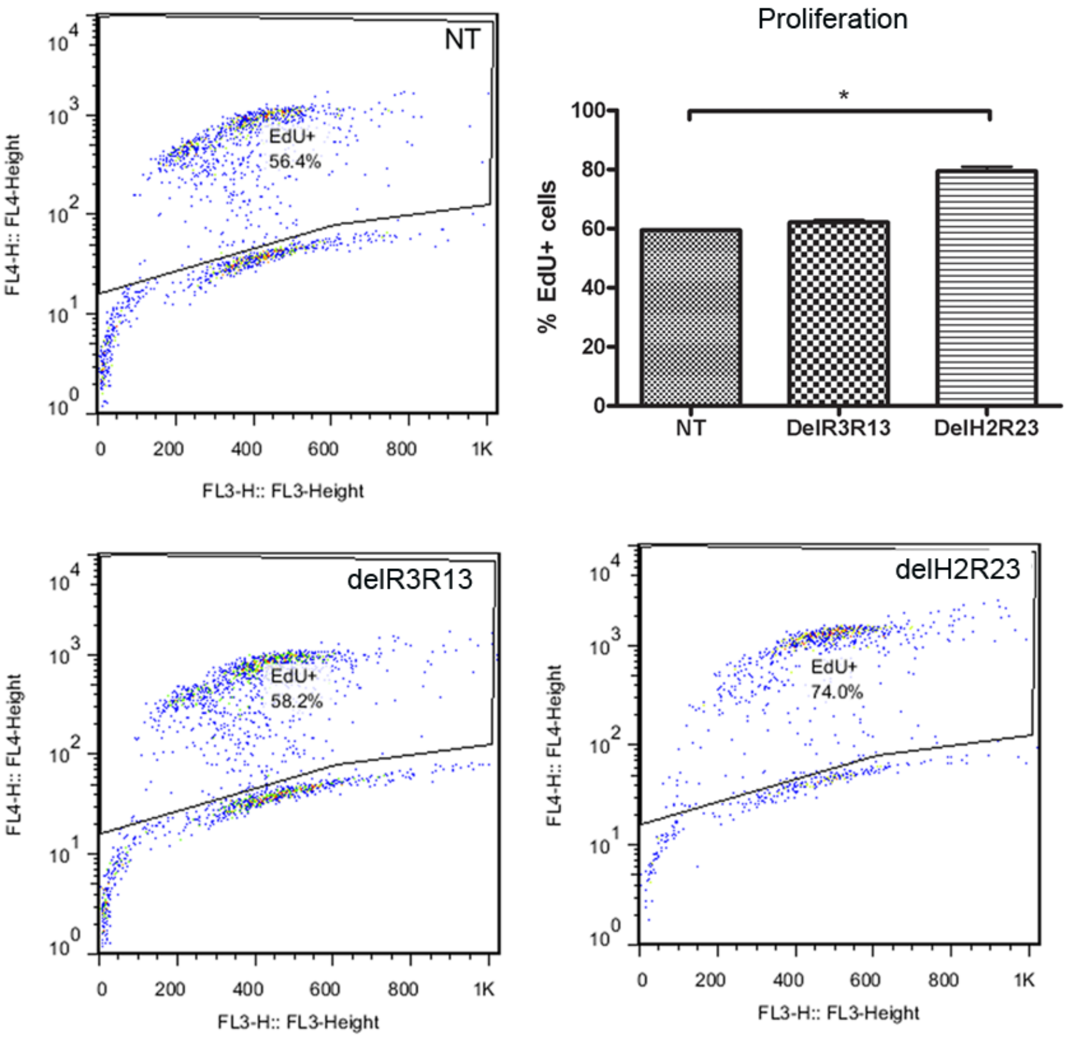

Figure S1. Effects on cell proliferation after cells were transduced with mini-dystrophin lentivirus driven by the human desmin promoter. Similar to cells transfected by SFFV-driven lentiviruses, mini-dystrophin ΔR3R13 has no effect on cell proliferation, while ΔH2R23 significantly increased the proliferation of human muscle-derived cells.
